# To Wait or Two Weeks: The Relationship Between Step 2 CK Scores and the Length of Dedicated Study Time Within a Longitudinal Interleaved Clerkship Curriculum

**DOI:** 10.7759/cureus.26599

**Published:** 2022-07-05

**Authors:** Sabrina Antonio, Rachel A Kracaw, Wynona Dizon, Edward Simanton

**Affiliations:** 1 Medical Education, Kirk Kerkorian School of Medicine at University of Nevada Las Vegas, Las Vegas, USA; 2 University of Nevada Las Vegas, Office of Medical Education, Las Vegas, USA

**Keywords:** dedicated study, step 2 performance, step 2 ck, core clerkships, longitudinal integrated clerkship, longitudinal interleaved clerkship, step 1 pass/fail, step 2

## Abstract

Introduction: Graduate medical education program directors report that United States Medical Licensing Examination (USMLE) Step 2 CK exam scores will likely have greater importance in the future selection of residents due to USMLE Step 1 transitioning to a pass/fail score as early as January 2022. With emphasis moving to the Step 2 exam, it is important to examine factors that maximize student Step 2 CK performance, such as third-year curriculum models and exam timing. This study analyzes whether or not Step 2 performance is affected by a specific length of dedicated study time within a Longitudinal Interleaved Clerkship (LInC) curriculum.

Methods: A regression model was used to predict Step 2 scores for 102 students using previous performance measures. Actual and predicted scores were compared to indicate which students overperformed or underperformed on Step 2. A t-test was used to compare the mean difference between predicted and actual performance of students who had two weeks or less of dedicated study time for Step 2 CK versus students who had a longer dedicated study period.

Results: Students who completed Step 2 with two weeks or less of dedicated study significantly overperformed (*t*(100)=2.06, *p*=0.042) on the exam (Mean=1.61, SD=9.21) compared to students who had more than two weeks of dedicated study (Mean=-1.67, SD=6.44) in a LInC curriculum.

Conclusion: Although studies of Step 2 preparation time have shown the importance of taking Step 2 soon after completion of clinical rotations, this study adds a specific timeframe. Our findings show that a dedicated study period of two weeks or less for Step 2 CK within a LInC curriculum is associated with better performance. This study was limited to a LInC curriculum and may not apply to other clinical year curricula.

## Introduction

Allopathic medical students must pass the United States Medical Licensing Examination (USMLE) for state licensure and specialty board certification. Students typically take Step 2 CK of the USMLE Exam following their clinical clerkships and after taking the National Board of Medical Examiners (NBME) Subject Exams in core clerkships. These subject exams typically include internal medicine, family medicine, pediatrics, surgery, obstetrics and gynecology (OBGYN), and psychiatry [1]. 

Step 2 is one of the factors considered for screening and selecting residency candidates during the application process and ultimately affects specialty match and location [2]. In the 2020 National Resident Matching Program (NRMP) survey, USMLE Step 2 is the third most cited factor that affects the selection of applicants to interview for residency programs, following USMLE Step 1 and letters of recommendation in the specialty [3]. With the implementation of pass/fail Step 1 scoring beginning as early as January 2022, more programs will require Step 2 scores to be submitted with applications in order to retain an objective measure for distinguishing applicants from one another [4-6]. In a survey of program directors from Accreditation Council for Graduate Medical Education (ACGME)-accredited residency programs in 30 specialties, 80.7% of program directors believe that Step 2 will have a greater influence on the residency application process [4]. In the competitive specialty of neurosurgery, academic faculty have reported that Step 2 would become “the new Step 1” for their program [5]. These findings highlight the increasing importance for evaluating approaches that may maximize students’ performance on Step 2.

Many studies have demonstrated that within a traditional clinical curriculum, Step 2 performance is inversely related to the time interval between the completion of core clinical clerkships and the Step 2 comprehensive exam [7-10]. Clerkship scheduling was examined with higher Step 2 scores found among students that had the surgery, OBGYN, and psychiatry clerkships closer to their test date [8-9]. Further, in a traditional block curriculum students who completed Step 2 earlier in June through August achieved the largest positive difference between their predicted and actual scores, while students who postponed the test until later saw decreases on average [10]. To date, no study has investigated specific time frames for optimizing Step 2 performance within a Longitudinal Interleaved Clerkship (LInC) curriculum.

Students in this study experienced a LInC model in which they progressed through a series of two-week rotations in various specialties. During this year-long experience, students returned to each of six primary specialties for another two-week rotation multiple times, adding up to a total of eight weeks (four two-week rotations) in surgery and internal medicine and three two-week rotations (six total weeks) in family medicine, OBGYN, pediatrics, and psychiatry. Students took all six NBME Subject Exams in one week at the midpoint of the LInC and again at the end of the curriculum. This unique type of clinical curriculum eliminates the bias from the order in which clerkships are taken.

This study aims to analyze whether students who had a dedicated study time of no longer than two weeks performed better than students who took more time to study for Step 2 within the LInC curriculum at the Kirk Kerkorian School of Medicine (KSOM) at the University of Nevada Las Vegas (UNLV). This information can have an impact on potential changes to medical school curricula and the advice provided to medical students regarding how to study and when to take Step 2.

## Materials and methods

KSOM is a newly accredited United States medical school that graduated its charter class in May 2021. The primary clinical year at KSOM is structured as a LInC model. In this curriculum, rotations last for two weeks before students move on to the next specialty. This approach is different from most Longitudinal Integrated Clerkship (LIC) models which traditionally use half-day experiences in each specialty. At KSOM, students are required to complete a total of eight weeks in surgery and internal medicine (four two-week rotations). All other core clerkships (family medicine, pediatrics, OBGYN, psychiatry) have three rotations each for a total of six weeks. Two selective rotations are also included in the LInC to allow for career exploration. To ensure longitudinal exposure to each specialty, rotations are scheduled so that every student has at least one rotation in each specialty in the first half of the LInC and again in the second half of the LInC. At the end of the LInC, students are provided with six weeks of independent study that can be used for Step 2 exam preparation, completion of missed clinical experiences, or optional clinical experiences. Students complete NBME Subject Exams in all six specialties over the course of four to five days (one school week) at the midpoint of the LInC. At the end of the LInC, students have the option to retake the NBME Subject Exams over six days so that they take only one subject exam per day. Students who passed an exam at the midpoint may opt out of the second round of exams; however, most students choose to take the exams again to improve their scores and to prepare for USMLE Step 2. The highest score of the two attempts is reported for the subject exam portion of students’ clerkship grades. Students are encouraged to take Step 2 soon after they have completed their second round of NBME subject exams.

At the time of this study, 106 students from the first two class cohorts at KSOM had taken Step 2. Four students who took Step 2 far outside the normal testing times were excluded from the study. This excluded students who either took Step 2 prior to the end of the LInC or waited more than four months after the second round of NBME subject exams before taking Step 2. In order to determine the relationship between Step 2 performance and the length of dedicated study time, de-identified student performance data was gathered from institutional databases in accordance with an approved IRB protocol (#1030906-1) and analyzed using SPSS Statistics version 26.0 (IBM Corp., Armonk, NY, USA). Mean performance was compared between students who took Step 2 within a two-week dedicated study period and students who took Step 2 after more than two weeks of dedicated study using an independent samples t-test. 

Since it is expected that stronger students would take Step 2 sooner than other students, a method was devised to control for this self-selection bias. A linear regression model was used to predict students’ Step 2 scores using previous performance measures. These performance measures included NBME subject exam scores, USMLE Step 1 score, mean exam score from the basic sciences curriculum, Medical College Admission Test (MCAT) scores, undergraduate grade point average (GPA), and undergraduate science GPA. The influence of these variables and the effectiveness of this prediction model were analyzed previously in the study "Predicting United States Medical Licensing Examination Step 2 Clinical Knowledge Scores from Previous Academic Performance Measures within a Longitudinal Interleaved Curriculum" [11]. Actual and predicted scores were compared to indicate which students overperformed or underperformed on Step 2. An independent samples t-test was used to compare the mean difference between predicted and actual performance of the two groups.

## Results

Data from 102 students (n=102) in the 2021 and 2022 graduating classes at KSOM were included in the sample. The group with two weeks or less of dedicated study time contained 54 students and the group that studied for more than two weeks contained 48 students. The raw mean score and standard deviation for students in each of the two groups are demonstrated in Table 1. The mean score for students who completed Step 2 within a two-week dedicated study period was 251.87 (SD 10.67) versus 240.81 (SD 15.59) for students who spent more than two weeks in dedicated study time. The pass rate was 100% in both groups.

**Table 1:** Means, standard deviations, and standard errors of the mean for actual scores of students who had a dedicated study period of two weeks or less and students who studied for more than two weeks

Length of Dedicated Study Time for Step 2 CK	Number of Students (n)	Mean + Standard Deviation (SD)
≤ 2 weeks	54	251.87 + 10.67
> 2 weeks	48	240.81 + 15.59

**Figure 1:**
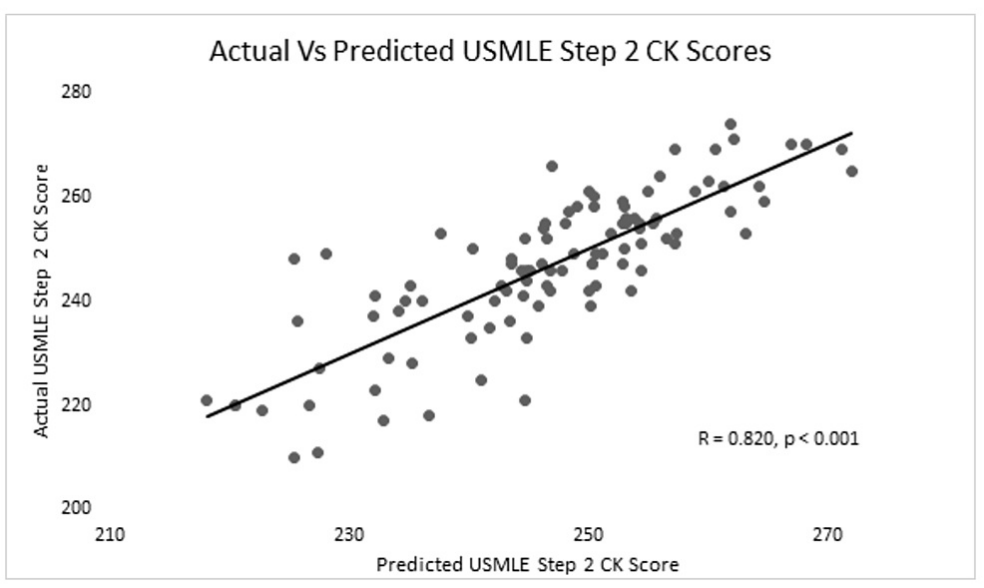
Graph of the actual versus predicted Step 2 scores

For the linear regression model used to predict Step 2 scores, the Pearson coefficient was R = 0.820 (p<0.001) with a standard error of estimate +/- 8.64. Figure 1 shows the linear regression model for actual versus predicted USMLE Step 2 scores.

Table 2 shows the data for the mean difference between actual and predicted Step 2 scores for each of the two groups analyzed. The group with a dedicated study period of no longer than two weeks (n=54) was associated with a positive average mean difference between actual and predicted scores (Mean=1.61, SD=9.21). By comparison, the group that devoted more than two weeks of dedicated study for Step 2 (n=48) was associated with a negative average mean difference between actual and predicted scores (Mean=-1.67, SD=6.44). 

**Table 2:** Means, standard deviations, and standard errors for the mean differences between actual and predicted Step 2 scores of the study groups

Length of Dedicated Study Time for Step 2 CK	Number of Students (n)	Mean Difference + Standard Deviation (SD)
≤ 2 weeks	54	1.61 + 9.21
> 2 weeks	48	-1.67 + 6.44

The 54 students who devoted no more than two weeks to Step 2 dedicated study time (Mean=1.61, SD=9.21) compared to the 48 students who studied longer (Mean=-1.67, SD=6.44) demonstrated a statistically significantly higher and more positive mean difference between actual and predicted Step 2 scores. A two-sample t-test was performed to compare the mean difference of actual vs predicted scores for the two study groups. The results of the t-test showed that the difference between the study groups was statistically significant t(100)=2.06, p=0.042.

## Discussion

This study found that students within a LInC curriculum who took Step 2 within a two-week dedicated study period strongly outperformed students who studied for more than two weeks. However, this finding can be attributed to self-selection and the potential presence of factors such as higher Step 1 scores associated with higher Step 2 scores [12-14]. After controlling for this self-selection bias, students that took USMLE Step 2 within two weeks of completing their NBME subject exams outperformed their predicted scores, while students who delayed Step 2 underperformed their predicted scores. 

The findings of this study are consistent with current literature which demonstrates it is better to take Step 2 sooner rather than later [7-10]. These results may influence the advice given to medical students regarding how to study and when to take their high-stakes, comprehensive Step 2 exam. With Step 1 transitioning to a pass/fail score as early as January 2022, the findings of this study will have great importance for advising students. Graduate Medical Education program directors and assistant program directors at residency programs across all specialties have voiced increased emphasis on Step 2. This affects the advice they will give to medical students entering future application cycles and how they will select applicants to interview [4-6]. 

Several limitations exist in this study. First, it was conducted at a single institution with a LInC curriculum and may not apply to other clinical year curricula. Second, only two class cohorts had taken Step 2 at KSOM when this study was conducted. This limited the sample size, and thus the overall power, of the study. Students’ academic performance may also have been influenced by the experience of attending a new medical school where there is less guidance from senior students and alumni. Third, the COVID-19 pandemic has affected the medical school experience of both cohorts included in the study [15-16]. Testing center closures caused changes and delays to the NBME Subject Exams and Step 2 test dates for the Class of 2021. The Class of 2022 had more virtual experiences due to a suspension of clinical rotations caused by a shortage of personal protective equipment available at the beginning of the pandemic. These factors likely influenced KSOM students’ study habits throughout their primary clinical year and required them to adjust to unique stressors associated with the pandemic. 

Future research is needed to understand the effects of specific timing on Step 2 performance in other clerkship curriculum models at other medical schools. This will strengthen the ability to recommend more specific time constraints that optimize medical student performance on Step 2 in various clerkship curriculum models.

## Conclusions

This is the first study to examine the relationship between Step 2 CK scores and a specific length of dedicated study time within a LInC curriculum. In the future, similar studies can be conducted for other types of clerkship curriculum models or at other medical schools that may implement the LInC format in their programs. Increasing emphasis on Step 2 scores following the transition of Step 1 to a pass/fail system beginning in January 2022 calls for additional studies and updated advising regarding methods to improve Step 2 scores.
